# Silencers of HTLV-1 and HTLV-2: the *pX*-encoded latency-maintenance factors

**DOI:** 10.1186/s12977-019-0487-9

**Published:** 2019-09-06

**Authors:** Robert Harrod

**Affiliations:** Laboratory of Molecular Virology, Department of Biological Sciences, and The Dedman College Center for Drug Discovery, Design & Delivery, Southern Methodist University, 6501 Airline Drive, 334-DLS, Dallas, TX 75275-0376 USA

**Keywords:** HTLV-1, HTLV-2, Tax, p13^II^, p30^II^, p28^II^, HBZ, APH-2, ATLL, HAM/TSP

## Abstract

Of the members of the primate T cell lymphotropic virus (PTLV) family, only the human T-cell leukemia virus type-1 (HTLV-1) causes disease in humans—as the etiological agent of adult T-cell leukemia/lymphoma (ATLL), HTLV-1-associated myelopathy/tropical spastic paraparesis (HAM/TSP), and other auto-inflammatory disorders. Despite having significant genomic organizational and structural similarities, the closely related human T-cell lymphotropic virus type-2 (HTLV-2) is considered apathogenic and has been linked with benign lymphoproliferation and mild neurological symptoms in certain infected patients. The silencing of proviral gene expression and maintenance of latency are central for the establishment of persistent infections in vivo. The conserved *pX* sequences of HTLV-1 and HTLV-2 encode several ancillary factors which have been shown to negatively regulate proviral gene expression, while simultaneously activating host cellular proliferative and pro-survival pathways. In particular, the ORF-II proteins, HTLV-1 p30^II^ and HTLV-2 p28^II^, suppress Tax-dependent transactivation from the viral promoter—whereas p30^II^ also inhibits PU.1-mediated inflammatory-signaling, differentially augments the expression of p53-regulated metabolic/pro-survival genes, and induces lymphoproliferation which could promote mitotic proviral replication. The ubiquitinated form of the HTLV-1 p13^II^ protein localizes to nuclear speckles and interferes with recruitment of the p300 coactivator by the viral transactivator Tax. Further, the antisense-encoded HTLV-1 HBZ and HTLV-2 APH-2 proteins and mRNAs negatively regulate Tax-dependent proviral gene expression and activate inflammatory signaling associated with enhanced T-cell lymphoproliferation. This review will summarize our current understanding of the pX latency-maintenance factors of HTLV-1 and HTLV-2 and discuss how these products may contribute to the differences in pathogenicity between the human PTLVs.

## Background

The primate T-cell lymphotropic virus (PTLV) family consists of the simian T-cell lymphotropic virus types 1–5 (STLV types 1–5) and human T-cell lymphotropic virus types 1–4 (HTLV types 1–4), which includes the human T-cell leukemia virus type-1 (HTLV-1) and the related human T-cell lymphotropic virus type-2 (HTLV-2, subtypes 2a, 2b, and 2d) [1–10]. The HTLV-1 is a delta oncoretrovirus that is endemic to tropical equatorial regions, including Southeast Asia (i.e., Japan, China, Taiwan, Malaysia, and the Philippines), Australia and Melanesia, Northern and Central Africa, the Middle East, Central and South America, and certain islands of the Caribbean (in particular, the FWI). Importantly, the HTLV-1 is considered to be an emerging health threat and has been identified in the indigenous populations in Australia and South America. The HTLV-1 infects dendritic cells, monocytes and CD4+ helper T-cells, and oncogenically transforms CD4+ T-cells and causes adult T-cell leukemia/lymphoma (ATLL)—an aggressive and often-fatal hematological malignancy that is poorly responsive to most anticancer treatments, in 3–5% of infected individuals. The HTLV-1 is also etiologically associated with a demyelinating neuro-inflammatory disease, known as HTLV-1-associated myelopathy/tropical spastic paraparesis (HAM/TSP), as well as other autoimmune/inflammatory disorders, including uveitis, rheumatoid arthritis, keratoconjunctivitis, infectious dermatitis, sicca syndrome, and Sjögren’s syndrome. There are currently approximately 10–20 million HTLV-1-infected individuals worldwide; and the virus is transmitted via infected lymphocytes present in blood/blood-products or body fluids through breastfeeding, sexual intercourse, blood transfusions, percutaneous injections, and IV-drug use with contaminated needles. The HTLV-2 was originally isolated from a patient with a rare benign form of hairy T-cell leukemia [9, 10] and, by contrast, this virus is generally considered to be apathogenic. The HTLV-2 infects B-cells and CD4+ and CD8+ T-cells, but preferentially induces oncogenic transformation in CD8+ T-cells in vitro [11–13]. Murphy et al. [14] have further suggested that HTLV-2 may be associated with HAM/TSP and other neurological symptoms in certain infected patients. Using a rabbit model of pathogenesis, combined with in vitro T-cell culture/immortalization studies, Kannian et al. [15] demonstrated that the HTLV-1 and HTLV-2 are comparably detected in both the CD4+ and CD8+ T-cell subpopulations as early as 1-week following the initial infection of experimental animals. Their findings further implied that the transformation tropism of these PTLVs (i.e., CD4+ T-cells for HTLV-1 and CD8+ T-cells for HTLV-2) is driven by the clonal expansion and selection of a transformed proviral cellular clone over a latency period of several decades, as occurs in HTLV-1+ ATLL patients [15].

**Fig. 1:**
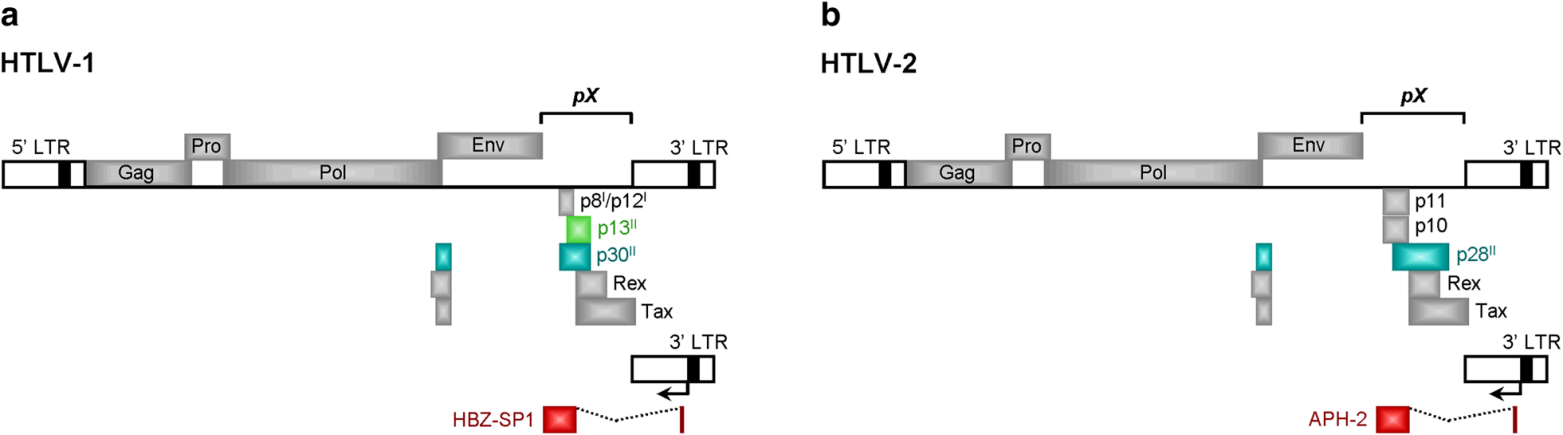
Diagrams of the HTLV-1 and HTLV-2 proviral genomes and their products. **a** The coding regions of the HTLV-1 genome are represented by filled boxes. The antisense HBZ-SP1 product is shown at the bottom with an arrow indicating its transcriptional initiation site from the 3′ LTR. **b** The HTLV-2 genome and its products. The coding region for the antisense-encoded APH-2 protein is indicated. The conserved *pX* nucleotide sequences are indicated in **a** and **b**. The *pX*-encoded latency factors discussed in this review are represented by colored boxes. *HBZ-SP1* HTLV-1 basic domain/leucine zipper-spliced-1 isoform, *APH-2* antisense protein of HTLV-2, *LTR* long terminal repeat

**Fig. 2:**
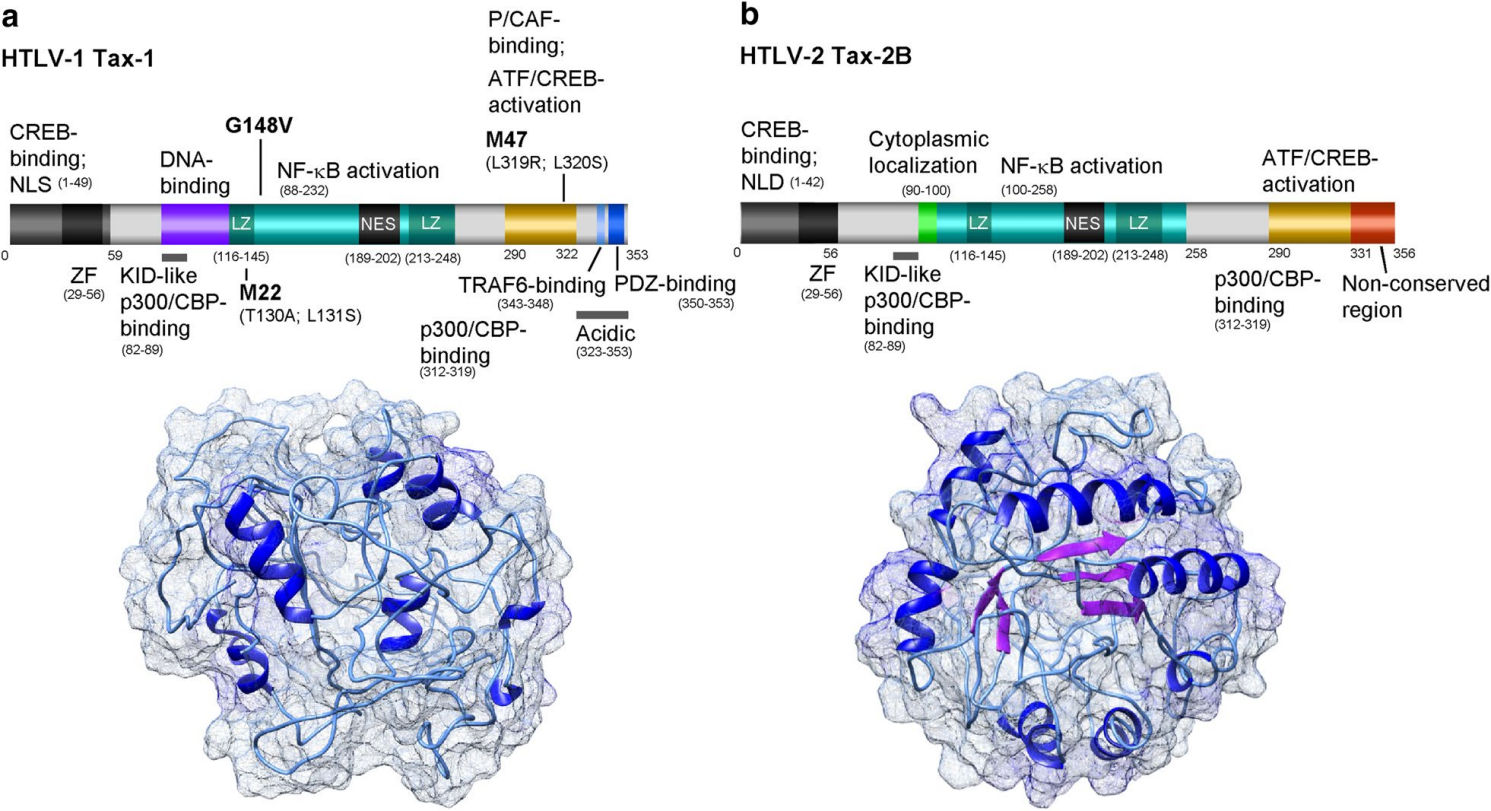
Functional domains and predicted structures of the HTLV-1 Tax-1 and HTLV-2B Tax-2 transactivator proteins. The predicted structures were generated using the I-TASSER computer algorithm (https://zhanglab.ccmb.med.umich.edu) and modeled using UCSF-Chimera. **a** Diagram of the HTLV-1 Tax protein and its functional domains. *NLS* nuclear localization signal, *NES* nuclear export sequence, *ZF* zinc finger motif, *LZ* leucine zipper region. The sites of the M22 (dimerization), G148 V (NF-κB transactivation), and M47 (activation domain) mutations are indicated. **b** The HTLV-2 Tax-2B protein and its conserved functional domains are shown. The unique C-terminal aa residues 331–356 are not present within the HTLV-1 Tax protein. *NLD* nuclear localization determinant

The HTLV-1 and HTLV-2 have complex genomes and encode several regulatory and accessory products within a highly-conserved 3′ nucleotide sequence, known as the *pX* region (Fig. 1a, b). The HTLV-1 *pX* sequence encodes the major transactivator protein, Tax-1 (Fig. 2a), the mRNA-splicing regulator, Rex, the open reading frame-I (ORF-I) products: p8^I^ and p12^I^, and the ORF-II products: p13^II^ and p30^II^. The HTLV-1 basic leucine zipper (bZIP) protein, HBZ, is encoded by the antisense (i.e., minus) strand of the integrated proviral DNA and the transcriptional initiation of *hbz* occurs from the 3′ LTR (Fig. 1a). By comparison, the HTLV-2 *pX* sequence encodes a Tax-2 transactivator (Fig. 2b) and Rex homologue, the accessory products p10 and p11, and the ORF-II p28^II^ protein (a functional synologue of HTLV-1 p30^II^). The antisense strand of HTLV-2 also encodes a nonconventional bZIP protein, APH-2, which is a synologue of HTLV-1 HBZ. These genes are expressed through alternative mRNA-splicing, and many (i.e., HTLV-1 HBZ, p30^II^ and p13^II^, and the HTLV-2 APH-2 and p28^II^ proteins; Fig. 1a, b) negatively regulate Tax-dependent transcriptional activity and maintain the latent silencing of proviral gene expression to promote viral persistence in vivo [16–21]. Indeed, numerous studies using animal models of pathogenesis have demonstrated that HTLV-1 HBZ, p30^II^, and p13^II^ and the HTLV-2 p28^II^ proteins are essential for immune-evasion, viral persistence and the maintenance of high proviral titers in vivo [22–26]. This review will discuss what is known about the expression of these *pX* latency-maintenance genes in HTLV-1 and HTLV-2-infected cell-lines, asymptomatic carriers, and ATLL and HAM/TSP clinical isolates, and how these factors could contribute to retroviral pathogenesis and disease progression.

## The antisense-encoded proteins, HTLV-1 HBZ and HTLV-2 APH-2

**Fig. 3:**
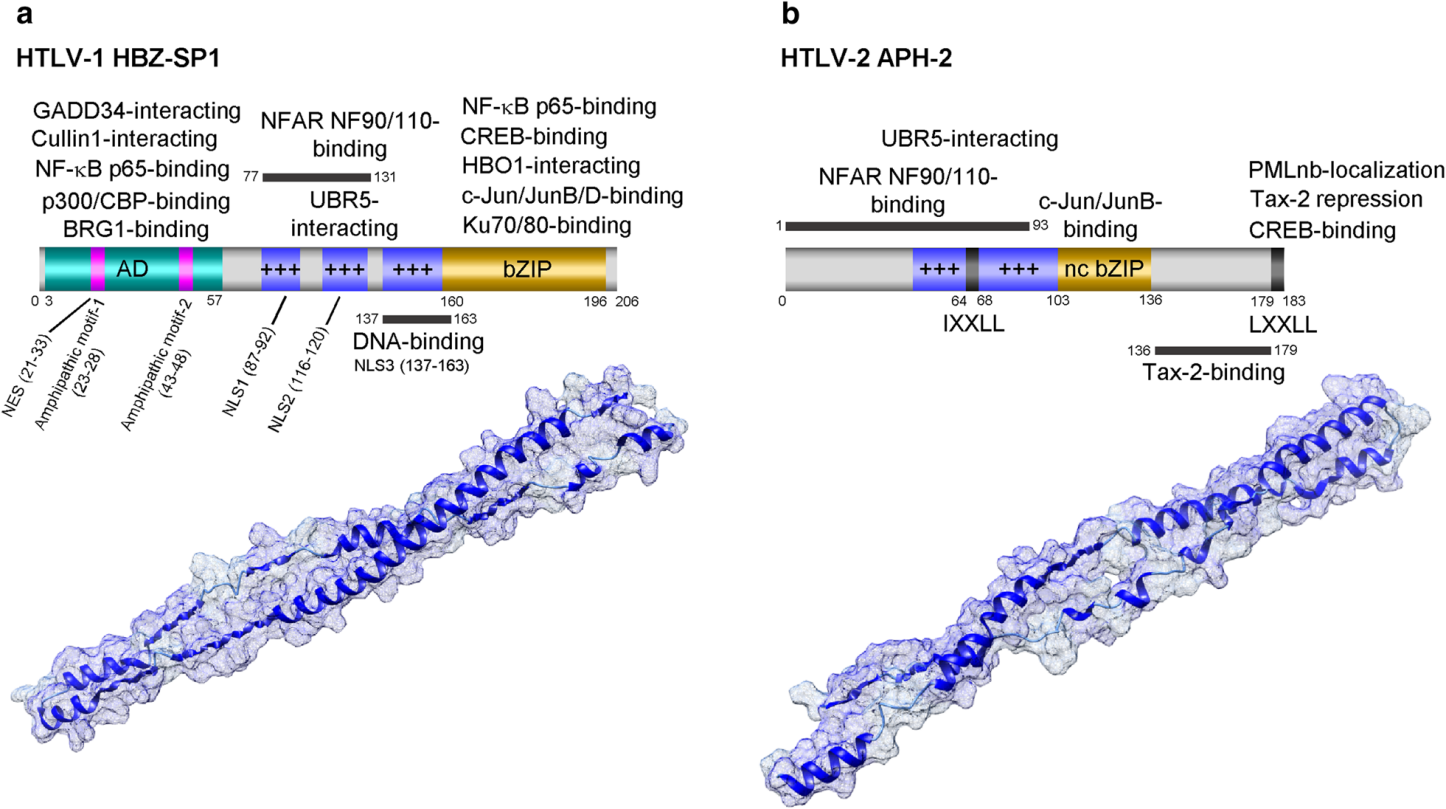
Functional domains and predicted structures of the antisense-encoded HTLV-1 HBZ and HTLV-2 APH-2 proteins. The predicted structures were generated using the I-TASSER algorithm and modeled using UCSF-Chimera. **a** Diagram and structure of the HTLV-1 HBZ-SP1 (spliced-1) isoform. The amphipathic helical motifs (1 and 2) which bind the KIX domains of p300/CBP are indicated. AD, activation domain; NES, nuclear export signal; NLS, nuclear localization signal. **b** The core IXXL and terminal LXXL modulatory sequences of the HTLV-2 APH-2 protein are indicated in the diagram. *nc bZIP* nonconventional basic domain/leucine zipper region, *NFAR NF90/NF110-binding* Nuclear factors associated with double-stranded RNA proteins NF90/NF110-binding region, *PLMnb-localization* PML nuclear body-localization/APH-2 stabilization domain. The basic regions in HBZ and APH-2 are represented by blue boxes with +++

The antisense strand of HTLV-1 encodes unspliced and alternatively-spliced transcripts, *hbz*, *hbz*-*sp1* and *hbz*-*sp2*, which code for different isoforms of a bZIP transcription factor: HBZ, HBZ-SP1 (spliced-1), and HBZ-SP2 (spliced-2), that negatively regulate proviral gene expression and modulate host lymphoproliferative signaling [27–33]. These transcripts are initiated from the 3′ LTR; and the *hbz*-*sp1* mRNA which codes for the most abundant isoform (HBZ-SP1) present in ATLL cells (Fig. 3a), includes exon 1 (nts 1–367) spliced to an acceptor site at position 1767 on the minus strand. The alternatively-spliced *hbz*-*sp2* mRNA which codes for the HBZ-SP2 protein has its first exon (nts 1–227) spliced to an acceptor site at position 1767 on the antisense RNA strand [28]. *Hbz* is the only viral gene detectable at every stage of infection in chronically-infected cell-lines and PBMCs derived from HTLV-1+ asymptomatic carriers, HAM/TSP, and ATLL patients. Despite the fact that the 5′ LTR is frequently inactivated in ATLL clinical isolates as a result of DNA hypermethylation or proviral deletions, the 3′ LTR and *hbz* mRNA and protein expression are usually intact, alluding to their pivotal roles in viral pathogenesis [34]. The HBZ protein is weakly immunogenic and not efficiently translated in ATLL lymphocytes. In 2014, Rowan et al. [35] demonstrated that autologous cytotoxic T-lymphocytes (CTLs), specific for an HBZ_26–34_ peptide epitope, effectively selected against HTLV-1-infected CD4+ T-cells which expressed a HLA-A*0201 major histocompatibility class I molecule that binds to HBZ-SP1 with high-affinity. These findings suggest a CD8+ cell-mediated immune-response could select for HTLV-1 proviral clones with reduced, steady-state levels of the antisense *hbz* products and intermittent *tax* expression in vivo.

## HBZ nuclear interactions and repression of Tax-dependent transcription

The antisense bZIP protein of HTLV-1, HBZ, antagonizes Tax-dependent viral gene expression through its nuclear interactions with the transcriptional coactivators p300/CBP and chromatin-remodeling components. The HBZ protein localizes in nuclear speckles and contains three nuclear localization signals, with NLS1 (aa 87–92) and NLS2 (aa 116–120) located within the two upstream basic domains and NLS3 (aa 137–163) in the DNA-binding region (Fig. 3a) [36, 37]. Mukai and Ohshima [38] have also shown that HBZ contains a nuclear export signal (NES), spanning amino acid (aa) residues 21–33 (Fig. 3a), and is shuttled to the cytoplasm in a CRM1-dependent manner where it binds and suppresses the growth-arrest and DNA-damage gene 34 (GADD34) to activate the mammalian target of rapamycin (mTOR) signaling pathway which could promote the growth and proliferation of HTLV-1-infected cells. Using LC–MS/MS analyses, Dissinger et al. [39] demonstrated that HBZ is posttranslationally modified by phosphorylation on serine residue S49, acetylation on lysines K66 and K155, and methylation on residues K35, K37, K181, and K186; however, these modifications did not appear to influence the stability or biological functions of the protein. HBZ negatively regulates Tax-dependent LTR transactivation and proviral gene expression by interacting with the bZIP domains of CREB/ATF-family transcription factors and inhibits their binding to the 21-bp-repeat *Tax*-*responsive elements* (TREs) of the HTLV-1 promoter [27, 30]. The N-terminal region of HBZ binds to the KIX domain of the transcriptional coactivators, p300/CREB-binding protein (p300/CBP), and interferes with the recruitment of p300/CBP by the viral transactivator to Tax/CREB/21-bp-repeat complexes on the 5′ LTR [40]. HBZ also inhibits the catalytic acetyltransferase activity of p300/CBP and prevented the acetylation of histone and non-histone targets, including the p53 tumor suppressor and NF-κB p65^RelA^ subunit [41, 42]. Moreover, Alasiri et al. [43] have demonstrated that HBZ interacts with the brahma-related gene 1 (BRG1) and BRG/hBRM-associated factor 200 (BAF200) components of the SWI/SNF (PBAF) chromatin-remodeling complexes and modulates their recruitment to Tax-containing 5′ LTR complexes to repress proviral gene expression. It is possible feedback interactions may coordinately regulate the expression of the proviral sense (plus-strand) and antisense (minus-strand) products, as Tax has been shown to transactivate the 3′LTR to drive *hbz* expression. The HBZ protein suppresses AP-1-dependent transcription through interactions with the bZIP factors, c-Jun and Jun B [44]. By contrast, HBZ activates JunD and stimulates the *human telomerase reverse transcriptase* (*hTERT*) gene promoter [45]. HBZ also preferentially induces the expression of the ∆JunD isoform through the repression of a ribosomal small subunit protein, RPS25, which promotes translational leaky-scanning past an upstream ORF and initiation on a downstream ORF [46]. This study further demonstrated that HBZ cooperates with the pro-proliferative ∆JunD isoform and enhanced its oncogenic colony-forming potential in vitro [46]. HBZ could also promote proviral latency through its interactions with the NF-κB p65^RelA^ subunit which inhibits p65^RelA^ DNA-binding and NF-κB transactivation [47]. Moreover, in 2011, Zhi et al. [48] reported that HBZ counters the cellular senescence and cytotoxicity associated with Tax-induced NF-κB hyper-activation in stable HeLa-G/FLAG-HBZ cell-lines that were transduced with adenoviral Tax expression vectors.

## Dynamic regulation of HTLV-1 gene expression by HBZ and Tax

The regulation of HTLV-1 proviral gene expression occurs through dynamic coordinate interactions between the sense and antisense *pX*-encoded products. In 2017, Billman et al. [49] used RNA-fluorescence in situ hybridization (RNA-FISH) to quantify the single-cell expression of *tax* and *hbz* transcripts within individual T-cell clones established from HTLV-1-infected patients. These findings demonstrated that *hbz* is not constantly expressed in every cell, but, rather, the *tax* and *hbz* mRNAs are produced in intermittent bursts—with *hbz* exhibiting a predominantly intranuclear localization and corresponding to cells in either the S or G2/M-phases of the cell-cycle [49]. Mahgoub et al. [50] further demonstrated that the viral transactivator Tax is persistently expressed at low levels in the HTLV-1-infected cell-line MT-1 and switches between ‘on’ and ‘off’ states within individual cells. *Tax* expression was important to protect these cells against apoptosis and delayed their transition into the G2/M-phase; and shRNA-knockdown of *tax* resulted in significant cellular cytotoxicity, suggesting that low levels of Tax are needed to promote the continuous survival of HTLV-1-infected leukemic cells in vivo. The *hbz* mRNA has been shown to indirectly increase levels of the Tax protein by inhibiting the expression of *pX*-*orfII*-*p30*^*II*^ transcripts [51]. Interestingly, Rushing et al. [52] reported that HBZ causes genotoxic stress, resulting in the accumulation of double-stranded DNA breaks, through its interactions with the Ku70/Ku80 subunits of DNA-PK and inhibition of the nonhomologous end-joining (NHEJ) repair pathway. It is likely a balance exists between HTLV-1 pX regulatory factors which may have ancillary roles in viral pathogenesis. Indeed, Hutchison et al. [53] have shown that the ORF-II p30^II^ protein cooperates with Tax and HBZ and countered their cytotoxicity due to oxidative stress, and enhanced the oncogenic potential of these viral proteins in vitro.

## Induction of T-cell lymphoproliferation by HBZ

The HBZ protein induces T-cell lymphoproliferation and enhances cellular survival through several different mechanisms. HBZ inhibits the classical Wnt signaling pathway through binding to lymphoid enhancer-binding factor 1 (LEF1), while also activating the noncanonical Wnt5a signaling pathway which could promote ATLL cell proliferation [54]. In 2013, Zhao et al. [55] demonstrated that HBZ interacts with the bZIP factor CCAAT/enhancer binding protein-alpha (C/EBPα) and inhibits its negative growth-suppressive functions in transfected Jurkat and 293T cells, in a Smad3-dependent manner. The HBZ protein also activates E2F-1-dependent transcription, associated with G1/S cell-cycle progression and apoptosis, through interactions with retinoblastoma protein (Rb)/E2F-1 complexes and displacement of the histone deacetylase, HDAC3 [56]. HBZ induces enhanced lymphoproliferation mediated by T-cell receptor (TCR) signaling, as a result of interfering with the recruitment of SHP-1/2 tyrosine phosphatases to inhibitory coreceptors, PD-1 and TIGIT, on the surfaces of HTLV-1-infected CD4+ T-cells [57]. Moreover, Forlani et al. [58] have shown that the HBZ protein exclusively localizes in the cytoplasm of cells isolated from HTLV-1+ asymptomatic carriers and HAM/TSP patients. The expression of *hbz* correlates with disease severity in HAM/TSP patients and could also potentially serve as a surrogate marker for therapy-responsiveness [59].

## Latency-maintenance and in vivo functions of HBZ

Although *hbz* is dispensable for the infection and immortalization of primary T-cells by HTLV-1 in vitro, it is required for viral persistence and the maintenance of a high proviral titer in vivo [22]. By inoculating rabbits with irradiated 729 B-cell-lines that contained HTLV-1 ACH proviral clones, expressing either wild-type HBZ or deletion mutants of HBZ (i.e., HTLV-1HBZ∆LZ or HTLV-1∆HBZ), Arnold et al. [22] demonstrated the antibody response against HTLV-1 antigens (p19^Gag^) and viral persistence in vivo as measured by quantitative RT-PCR is dependent upon *hbz* gene expression. A study of the kinetics of viral gene expression demonstrated the levels of *tax/rex*, *gag/pol*, and *env* mRNAs decreased and inversely correlated with higher levels of the *hbz* transcripts in infected rabbits [60]. Rende et al. [61] have reported that 90% of the *hbz* mRNAs are compartmentalized and sequestered in the nuclei of cultured HTLV-1-infected T-cell clones established from ATLL and HAM/TSP patients. Valeri et al. [23] have further shown that *hbz* is required for viral persistence in rabbits and Rhesus macaques inoculated with lethally-irradiated 729 B-cell-lines expressing the wild-type HTLV-1 provirus or mutants ablated for *hbz* expression (or other ORF-I and ORF-II products), with genetic reversion to the wild-type sequence observed in 3 out of 4 seropositive macaques inoculated with the HBZ-knockout mutant. The *hbz* mRNA and HBZ protein differentially promote T-cell activation, lymphoproliferation and cell survival [33, 34]. The HBZ protein induces cellular apoptosis, whereas the *hbz* mRNA protects against programmed cell-death and induces the expression of cell-cycle regulatory and anti-apoptotic genes (e.g., *survivin*) in transduced primary murine T-cells. Both the HBZ protein and mRNA were able to induce T-cell lymphoproliferation and aberrant S-phase entry [33]. Small-interfering or short hairpin RNAs that inhibit *hbz* expression blocked the in vitro proliferation of HTLV-1-transformed T-cell-lines and ATLL cells [31, 34]. Arnold et al. [31] also demonstrated that HTLV-1-transformed SLB1 lymphoma cells, transduced with shRNA lentiviral vectors targeted against *hbz*, exhibit reduced cell proliferation, tumorigenesis, and secondary tissue infiltration in engrafted NOD/scid^γchain−/−^ animals. The *hbz* mRNA posttranscriptionally increased the expression of oncogenic microRNAs, miR17 and miR21, in CD4+ T-cell clones established from HTLV-1-infected HAM/TSP patients [62]. Importantly, the expression of HBZ in the CD4+ T-cells of *hbz*-transgenic mice resulted in the formation of skin and lung lesions associated with systemic inflammation and lymphocyte infiltration [32, 63]. Many of these animals also developed T-cell lymphomas following a prolonged latency. The HBZ protein interacts with FoxP3/NFAT transcription complexes and inhibited FoxP3-dependent immunosuppressive-signaling in CD4+ T_reg_ cells which resulted in increased inflammation [32]. In 2011, Zhao et al. [64] demonstrated that HBZ forms ternary complexes with Smad3 and the p300 transcriptional coactivator and enhances transforming growth factor-beta (TGF-β) signaling, associated with the increased expression of FoxP3 and conversion of HTLV-1-infected CD4+ cells into T_reg_ cells. Esser et al. [65] have further shown that *granzyme B* promoter-*hbz* transgenic mice developed CD45+ mixed-cellular tumors, with enlargement of the spleen, elevated white blood cell counts, and osteolytic bone metastases associated with the increased expression of inflammatory cytokines and factors involved in hypercalcemia, including RANKL, PTHrP and DKK1.

## The HTLV-2 counterpart of HBZ, APH-2

The antisense protein of HTLV-2, or APH-2, is a functional synologue of the HTLV-1 HBZ factor and is generated through alternative mRNA-splicing which uses a donor site at position 8544 and a splice acceptor site at position 7173 on the antisense strand of the pH6neo molecular clone of HTLV-2 [66]. APH-2 is a nuclear protein comprised of 183 aa residues and contains two core modulatory aliphatic sequences: IXXLL (aa 64–68) and LXXLL (aa 179–183), and a basic region located upstream from a noncanonical bZIP motif (Fig. 3b) [66]. In 2009, Halin et al. [66] demonstrated that APH-2 interacts with the CREB transcription factor and inhibits Tax-2-mediated transactivation from the HTLV-2 LTR in luciferase reporter assays and represses proviral gene expression (p19^Gag^) by the pH6neo HTLV-2 clone, suggesting APH-2 antagonizes Tax-2 functions and promotes viral latency in vivo. The repression of Tax-2-dependent transactivation and binding to CREB were dependent upon the C-terminal modulatory sequence, LXXLL, of APH-2 [67]. Unlike HBZ, however, the APH-2 protein does not interact with the p300/CBP transcriptional coactivators [66]. The *aph*-*2* mRNA is constitutively expressed in chronically-infected cell-lines and PBMCs derived from HTLV-2-infected carriers [66]. Although the *aph*-*2* mRNA levels coincided with the proviral loads in HTLV-2-infected patients, neither the *aph*-*2* mRNA nor APH-2 protein were able to induce lymphoproliferation in vitro [68]. Bender et al. [69] have reported that the majority of *aph*-*2* transcripts are sequestered in the nuclei of HTLV-2-infected cells, similar to the subcellular compartmentalization of *hbz* mRNAs. In 2012, Yin et al. [67] demonstrated that APH-2 is dispensable for viral infectivity and the immortalization of primary T-cells in vitro; and rabbits inoculated with an irradiated 729 B-cell-line that contained an HTLV-2 proviral deletion mutant of APH-2 (∆Aph-2) exhibited increased antibody titers and proviral loads, as compared to animals inoculated with 729/wild-type HTLV-2 clones. These findings suggest HBZ and APH-2 are functionally divergent for the maintenance of viral persistence in vivo. Both HBZ and APH-2 inhibit NF-κB p65^RelA^-dependent transcriptional activation; however, by contrast, APH-2 does not augment TGF-β signaling [70]. The HBZ protein was discovered to be significantly more stable than APH-2 in half-life assays using cycloheximide-treated cells [70]. While both HBZ and APH-2 were shown to interact with the E3 ubiquitin ligase, UBR5, only HBZ was stabilized by knocking down UBR5 expression [71]. Dubuisson et al. [72] have further demonstrated that the APH-2 protein is shuttled to PML nuclear bodies, in a manner dependent upon APH-2-SUMOylation, where it is degraded by the proteasome. The noncanonical bZIP domain of APH-2 interacts with c-Jun and JunB; and APH-2 activates AP-1-dependent transcription [73]. Marban et al. [73] have also demonstrated that the C-terminal region of APH-2 binds to Tax-2 and inhibits Tax-dependent Ap-1 transactivation in cotransfected 293T cells. The central domain of HBZ and aa residues 1–93 of APH-2 interact with the nuclear factors associated with double-stranded RNA (NFAR) proteins, NF90 and NF110 (Fig. 3a, b), which are involved in innate immunity as targets of PKR-activation and, in addition, enhance Tax-dependent transactivation from the viral LTR and transcriptionally activate the *survivin* gene associated with the cellular anti-apoptotic response [74]. The siRNA-knockdown of NFAR did not significantly affect the ability of APH-2 to repress Tax-2-dependent LTR transactivation; and APH-2 inhibited *survivin* promoter transactivation by the NFAR NF110a [74]. These results suggest that HBZ and APH-2 interact with the NFARs to modulate viral gene expression and latency, as well as host innate-immunity and anti-apoptotic signals.

## The ORF-II latency-maintenance factors, HTLV-1 p30^II^ and HTLV-2 p28^II^

The ORF-II proteins which suppress the expression of proviral antigens represent an understudied area in the HTLV field, yet—in the light of recent evidence, there is reason to believe these factors may have key ancillary functions that could provide clues into the different pathogenic properties of the human PTLVs. The conserved *pX* regions of HTLV-1 and HTLV-2 encode the ORF-II products: p30^II^ and p28^II^, respectively, through alternative mRNA-splicing, which negatively regulate the Tax-dependent expression of viral antigens [16–20, 75] and are required for the maintenance of proviral latency and persistence in vivo [23, 24, 26]. In this regard, they are considered functional synologues [17]. However, mounting evidence indicates that p30^II^ and p28^II^ differ significantly in their capacity to modulate host signaling pathways and cooperate with other viral and cellular oncoproteins and, therefore, these factors are likely to have divergent roles in pathogenesis [18, 19, 53, 76–86]. This section will highlight the major similarities and differences between these ORF-II products, and discuss how they may contribute to mitotic proviral replication, T-cell immortalization, and the establishment and progression of neoplastic disease.

## Expression of the ORF-II products in HTLV-infected cell-lines, asymptomatic carriers, and ATLL and HAM/TSP patients

**Fig. 4:**
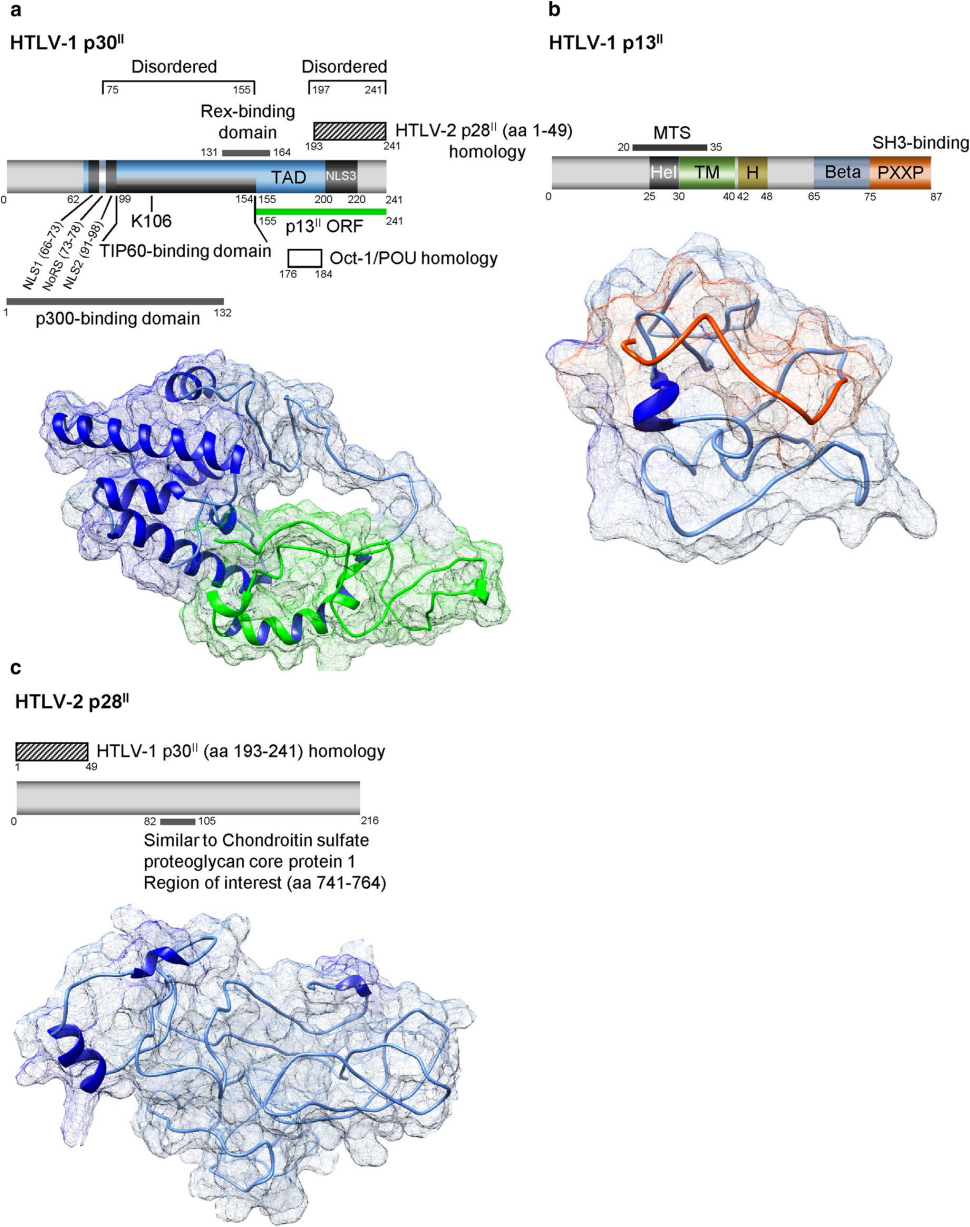
Functional domains and predicted structures of the HTLV-1 p30^II^, p13^II^, and HTLV-2 p28^II^ proteins. The predicted structures were generated using the I-TASSER algorithm and modeled using UCSF-Chimera. **a** Diagram and structure of HTLV-1 p30^II^. TAD, transcriptional activation domain; NLS, nuclear localization signal; NoRS, nucleolar retention sequence. **b** The p13^II^ aa sequence overlaps with the C-terminal region of HTLV-1 p30^II^ (aa 155–241) and is colored green in the p30^II^ structure (lower panel, **a**). The SH3-binding domain (aa 75–87) of HTLV-1 p13^II^ which contains a PXXP motif is colored orange in the diagram and in the modeled structure (**b**). MTS, mitochondrial targeting sequence; Hel, helical region; TM, transmembrane domain; H, flexible hinge region; Beta, predicted beta-sheet secondary structure. **c** The amino-terminus of the HTLV-2 p28^II^ protein (aa 1–49) shares 78% sequence homology with aa residues 193–241 of HTLV-1 p30^II^. A region of 58% similarity with a peptide sequence of the Chondroitin sulfate proteoglycan core protein 1 (aa 741–764) is also indicated

The HTLV-1 p30^II^ protein (also known as Tax-ORFII, or Tof-II) is comprised of 241 aa residues, contains arginine and serine/threonine-rich regions, and shares aa sequence similarities with the Oct-1/Pit-1/POU-family of homeodomain transcription factors (Fig. 4a) [87–89]. The C-terminus of p30^II^ (aa residues 155–241) also corresponds with the reading frame for the p13^II^ protein sequence (Fig. 4b) [88, 90–92]. The related HTLV-2 synologue, p28^II^, is comprised of 216 aa residues; and a peptide sequence (aa residues 1–49) within its N-terminus shares 78% sequence homology with residues 193–241 of HTLV-1 p30^II^ [17, 93]. However, there are no other sequence or structural similarities between these proteins outside of this region (Fig. 4c). Although p30^II^ has been reported to contain two intrinsically disordered sequences, spanning aa residues 75–155 and 197–241 [94], the p30^II^ protein is predicted to contain at least five alpha-helices which may contribute to its unique biological functions and molecular interactions (Fig. 4a). The HTLV-2 p28^II^ protein is predicted to be largely unstructured and exists as random coils, and little is known about the specific regions of p28^II^ that mediate its interactions with cellular factors (Fig. 4c). An NCBI-BLAST analysis identified a region (aa residues 82–105) that shares 58% similarity with aa residues 741–764 of the human chondroitin sulfate proteoglycan core protein 1, although the functional relevance of this sequence, if any, remains to be determined. The HTLV-1 *pX*-*orfII* mRNA which codes for p30^II^ is generated through alternative-splicing that includes exon 1 (nts 1–119) with exon 2 (nts 4641–4831) spliced to an acceptor site at position 6478 of the downstream *pX* sequence that is also used for the bicistronic *pX*-*tax/rex* mRNA [90, 95]. The alternatively-spliced *pX* mRNAs, including *pX*-*orfII*-*p30*^*II*^, have been detected by RT-PCR in cultured HTLV-1-infected T-cell-lines and primary uncultured ATLL clinical isolates, as well as in cells from asymptomatic HTLV-1-infected carriers [87, 90, 96]. In 2003, Princler et al. [95] demonstrated that the *pX*-*orfII* mRNA is expressed in chronically-infected T-cell-lines; and Cereseto et al. [97] have detected the *pX*-*orfII* mRNA in HTLV-1-transformed T-cell-lines, PBMCs from HTLV-1-infected carriers, and cells isolated from HAM/TSP patients using a non-PCR-based ribonuclease-protection assay. The HTLV-2 alternatively-spliced *pX*-*orfII*-*p28*^*II*^ mRNA, which is generated by splicing exon 1 (nts 316–449) to an acceptor site at position 6944 of the *pX* sequence, has been detected in the chronically-infected MoT cell-line by RT-PCR analysis [93]. Further, Pique et al. [91] have isolated CD8+ cytotoxic T-lymphocytes (CTLs) that specifically target the ORF-II p30^II^ and p13^II^ peptides from HTLV-1-infected carriers, HAM/TSP and ATLL patients, suggesting these proteins are chronically expressed and could contribute to the establishment of persistent infections in vivo as well as viral pathogenesis.

## In vivo requirement for the ORF-II latency-maintenance factors of HTLV-1 and HTLV-2

The functional roles of the ORF-II p30^II^ and p28^II^ proteins can be divided into: (a) the establishment of early-stage latency and viral persistence in vivo, and (b) their interactions with host proliferative-signaling pathways and cooperation with other viral (i.e., Tax and HBZ) and cellular factors to drive mitotic proviral replication. The HTLV-1 p30^II^ protein contains three putative nuclear localization signals (NLS1/2/3) as well as a nucleolar retention sequence (NoRS; Fig. 4a) and is primarily nuclear and/or nucleolar in its localization, although it is also frequently detectable in the cytoplasm [16, 20, 53, 84, 86, 88, 98]. The HTLV-2 p28^II^ protein is predominantly nuclear in its subcellular distribution [16, 98]. Using an established rabbit model of pathogenesis, Bartoe et al. [24] demonstrated that *pX*-*orfII* products are essential for the maintenance of a high proviral titer in experimental animals inoculated with PBMCs that contained either a wild-type molecular clone of HTLV-1 ACH.1, or an ACH.30^II^/13^II^.1 mutant defective for the expression of ORF-II proteins. While the *pX*-*orfII* products are widely thought to be dispensable for viral infectivity and the immortalization of primary T-cells in vitro [99, 100], the data presented in Table 1 of Robek et al. [100] indicate that the ACH.p30^II^ mutant exhibited a 50% reduced capacity to immortalize T-cells in in vitro co-culture assays, suggesting p30^II^ may contribute to HTLV-1-induced leukemogenesis. Indeed, Romeo et al. [86] have demonstrated that lentiviral p30^II^ induced the long-term proliferation beyond crisis (> 4 months) of transduced human PBMCs selected on blasticidin and cultured in the presence of recombinant interleukin-2 (IL-2), although these transiently-amplified clones were observed to undergo a second crisis at around 7 months and it is presumed that other viral and/or cellular factors are necessary for T-cell immortalization [86]. By contrast, a p28^II^-defective HTLV-2 mutant provirus, derived from the pH6neo molecular clone, exhibited viral infectivity and immortalized T-cells in vitro similar to the wild-type virus, but failed to promote proviral replication and T-cell survival in vivo in a rabbit model of HTLV-2 pathogenesis [26]. A study of the kinetics of HTLV-1 gene expression in cultured PBMCs isolated from ATLL and HAM/TSP patients, using splice-site specific quantitative RT-PCR analysis, revealed two-phase kinetics in the ATLL cells where the levels of *pX*-*tax/rex* mRNA were inversely correlated with the expression of the other *pX*-*orfII*, *pX*-*orfI*, and *hbz*-*sp1* transcripts [61]. A similar study which used 293T cells transiently transfected with the HTLV-1 ACHneo proviral clone failed to detect significant expression of the *pX*-*orfII* mRNA in vitro [60]. By contrast, in 2012, Bender et al. [69] investigated the kinetics of HTLV-2 gene expression and reported the *pX*-*tax/rex* and *pX*-*orfII*-*p28*^*II*^ transcripts were detected at comparable levels in the chronically-infected cell lines, MoT and BJAB-Gu, and in PBMCs isolated from 2 out of 3 HTLV-2-infected patients. The HTLV-1 p30^II^ protein has been shown to promote aberrant S-phase entry and lymphoproliferation, and induces the expression of T-cell activation and pro-survival genes [53, 77, 80, 84–86, 101]. In 2011, Anupam et al. [80] demonstrated that lentiviral p30^II^ enhanced the survival of transduced 293T and Jurkat T-cells, associated with p30^II^-interactions with the ataxia telangiectasia mutated (ATM) and REGγ proteins. A follow-up study by Doueiri et al. [82] demonstrated that an S-tagged p30^II^ protein interacts with the nuclear 20S proteasome activator REGγ. This study combined biochemical affinity purification with mass spectrometry analysis and identified several unique binding partners and three common interacting factors (i.e., protein arginine methyltransferase 5, hnRNP K, and large ribosomal subunit protein L8) that associate with the HTLV-1 p30^II^ and HTLV-2 p28^II^ proteins [82].

## Latency and the repression of Tax-dependent proviral gene expression by p30^II^ and p28^II^

The HTLV-1 p30^II^ and HTLV-2 p28^II^ proteins negatively regulate proviral gene expression and function as latency-maintenance factors which could help HTLV-infected cells evade host immune-surveillance pathways for the establishment of persistent infections in vivo. In 2000, Zhang et al. [19] used Gal4-p30^II^ fusion constructs and luciferase reporter assays and demonstrated that p30^II^ contains a functional transcriptional activation domain (Fig. 4a), and that p30^II^ differentially induces CREB-dependent transcription from the 21-bp repeat TREs in the HTLV-1 promoter, yet represses CREB-dependent transactivation from cellular *CREB*-*responsive elements* (CREs). The p30^II^ protein binds to the KIX domain of the transcriptional coactivators, p300/CBP, and competes against the viral transactivator Tax for the recruitment of p300/CBP to CREB/21-bp-repeat TRE complexes on the HTLV-1 promoter and represses the expression of viral antigens [18]. The transcriptional repression of the HTLV-1 5′ LTR by p30^II^ was dependent upon a single lysine residue at position K106 within the p30^II^ protein (Fig. 4a), and required p300-binding and the catalytic acetyltransferase domain of the p300 coactivator [20]. Interestingly, Datta et al. [76] have shown that p30^II^ interacts with the Ets domain of the PU.1 transcription factor, inhibits its DNA-binding, and represses the PU.1-dependent expression and activation of Toll-like receptor-4 (TLR4) in transfected cells. The inhibitory effect of p30^II^ upon PU.1-dependent transcriptional activation was countered through overexpression of the p300 coactivator. p30^II^ also inhibited the pro-inflammatory cytokines, MCP-1, TNF-α, and IL-8, and increased the release of the anti-inflammatory factor IL-10 following the stimulation of TLR4 in THP-1 monocytic cells with lipopolysaccharide, suggesting p30^II^ might interfere with adaptive immunological signaling in the early stages of viral pathogenesis [76]. These findings were supported in a study by Fenizia et al. [83] which demonstrated that p30^II^ inhibits the expression of interferon-responsive genes by interfering with the PU.1-dependent expression of TLR4 in THP-1 monocytes and dendritic cells. The inhibition of interferon-responsive signaling and innate immunity by p30^II^ could contribute to the early-stage establishment of infection and viral persistence in vivo. Valeri et al. [23] have also shown that p30^II^ is required for the productive infection of human dendritic cells by HTLV-1, and further demonstrated that Rhesus macaques inoculated with an irradiated 729 B-cell-line, containing an ACH.p30-knockout mutant ablated for *p30*^*II*^ expression, either failed to seroconvert or exhibited genetic reversion to the wild-type ACH sequence.

In 2004, Nicot et al. [16] reported that p30^II^ posttranscriptionally inhibits the nuclear export of the doubly-spliced bicistronic *pX*-*tax/rex* mRNA and negatively regulates HTLV-1 gene expression. For these studies, 293T cells were cotransfected with an HTLV-1 molecular clone, p-BST, and an expression construct for p30^II^ and the inhibition of viral gene expression was demonstrated by Anti-p19^Gag^ ELISAs and the nuclear accumulation of *pX*-*tax/rex* transcripts was detected with RT-PCR. This study further demonstrated that p30^II^ was associated with the splice-junction of the *pX*-*tax/rex* mRNA using biotinylated RNA-precipitation experiments, and that lentiviral p30^II^ negatively regulated proviral gene expression in transduced HTLV-1-transformed T-cell-lines (i.e., MT-2, C91PL, and HUT-102) [16]. Ghorbel et al. [98] have shown the nuclear/nucleolar retention of p30^II^ is dependent upon its interactions with large ribosomal subunit L18a within cell nucleoli. Younis et al. [17] subsequently demonstrated that both p30^II^ and the synologue p28^II^ posttranscriptionally inhibit proviral gene expression from molecular clones of HTLV-1 or HTLV-2. The HTLV-2 p28^II^ (AU1-tagged) protein was immunoprecipitated bound to *pX*-*tax/rex* mRNA complexes in vivo; and p28^II^ resulted in increased nuclear sequestration of the *pX*-*tax/rex* mRNA as detected by RT-PCR [17]. Interestingly, both p30^II^ and p28^II^ are recruited to their respective RNA targets through co-transcriptional interactions with the retroviral transactivator protein Tax on the promoters of the HTLV-1 and HTLV-2 proviruses [102]. In 2006, using chromatin-immunoprecipitation analyses, Younis et al. [102] demonstrated that p30^II^ and p28^II^ interact with Tax-1 or Tax-2 and are recruited and travel with RNA Polymerase II-containing transcriptional elongation complexes until they reach their nascent RNA targets. Using confocal microscopy and biochemical glutathione-S-transferase (GST)-pull-downs, Baydoun et al. [103] have shown that p30^II^ interacts with the retroviral splicing regulator, Rex, and sequesters Rex/CRM1 complexes in nucleoli in cotransfected COS and 293T cells. However, this somewhat contradicts findings by Sinha-Datta et al. [104] which demonstrated that aa residues 131–164 of p30^II^ interact with Rex complexed with the *Rex*-*responsive RNA element* (RxRE) of *tax/rex* mRNA transcripts, but this interaction did not interfere with the shuttling of Rex/CRM1/mRNA complexes out of the nucleus. The regulation of HTLV-1 gene expression and latency by Tax, HBZ, and p30^II^ (or HTLV-2 gene expression by Tax-2, Aph-2, and p28^II^) is orchestrated by coordinate and dynamic molecular interactions at the transcriptional and posttranscriptional levels. Choudhary and Ratner [51] have also shown that the *hbz*-*sp1* anti-sense mRNA indirectly increases expression of the Tax transactivator from a molecular clone of HTLV-1 by reducing the expression of the *pX*-*orfII*-*p30*^*II*^ mRNA, which lends additional complexity to the control of proviral gene expression by pX products.

## Activation of cellular pro-survival and proliferative signals by HTLV-1 p30^II^, but not p28^II^

Viruses that induce latent infections replicate their genomes by inducing mitotic host-cell division and proliferation. The p30^II^ protein of HTLV-1 helps to accomplish this by activating cellular oncogenic and pro-survival pathways through its molecular interactions with the TIP60 acetyltransferase—a transcriptional cofactor for both c-Myc and p53. In 2005, Awasthi et al. [84] demonstrated that p30^II^ cooperates with the c-Myc oncoprotein, induces aberrant lymphoproliferation in Molt-4 T-cells, and enhances the oncogenic potential of c-Myc in cellular transformation/foci-formation assays using human fibroblasts. Mechanistically, aa residues 99–154 of p30^II^ were shown to interact with the MYST-family acetyltransferase TIP60, using biochemical GST-pull-downs and co-immunoprecipitation experiments (Fig. 4a); and the p30^II^ protein was present in c-Myc/TIP60-containing NuA4 transcriptional activation complexes (together with the scaffold subunit TRRAP/p434, hGCN5, and the ATP-dependent DNA-helicases TIP48/TIP49 [105]) recruited to E-box enhancer elements within the *cyclin D2* gene promoter [84]. p30^II^ transcriptionally activated the *cyclin D2* promoter as well as a minimal M4-*tk*-luciferase reporter construct that contains four tandem E-box elements [84, 85]. Using dominant-negative mutants of TIP60 and TRRAP, it was found that the cooperation and oncogenic transformation by p30^II^/c-Myc were dependent upon the TRRAP transcriptional cofactor and the catalytic acetyltransferase domain of TIP60 [84]. At least three studies have examined the global gene expression profiles of cells containing HTLV-1 p30^II^ [78, 84, 101]. In 2005, Awasthi et al. [84] performed Affymetrix U133-Plus microarray gene expression analyses which included a dominant-negative acetyltransferase-defective mutant of TIP60, and identified 250 target genes that were transcriptionally activated or repressed by p30^II^ in a TIP60-dependent or -independent manner. In a follow-up study, Romeo et al. [85] used the HO15.19 *myc*-null rat fibroblast cell-line, together with various acetylation-defective Lys → Arg substitution mutants of c-Myc, and demonstrated that oncogenic cellular transformation by p30^II^/c-Myc required acetylation of the c-Myc oncoprotein and that p30^II^ co-immunoprecipitated with acetylated c-Myc [85]. The oncogenic foci-formation by p30^II^/c-Myc was dependent upon the acetyltransferase domains of TIP60, p300, and PCAF; however, this study did not determine which acetyltransferase(s) was primarily responsible for acetylation of the c-Myc oncoprotein [85]. The *p53* tumor suppressor is a downstream target of c-Myc; and acute and lymphoma-stage ATLL clinical isolates frequently contain c-Myc overexpression and elevated levels of wild-type p53 [106–108]. Intriguingly, the *p53* gene is rarely mutated in HTLV-1-infected ATLL patient samples [109–112], which leads to speculation that p53-dependent gene expression may somehow contribute to retroviral pathogenesis. Several studies have demonstrated that TIP60-mediated acetylation of the p53 protein on lysine residue K120 differentially regulates the induction of p53-dependent pro-apoptotic versus pro-survival/growth-regulatory genes [113–115]. In 2018, Romeo et al. [86] and Hutchison et al. [53] demonstrated that the HTLV-1 p30^II^ protein induces p53 and inhibits TIP60-mediated K120-acetylation of p53, and transcriptionally activates the expression of p53-regulated pro-survival genes. Most notably, lentiviral p30^II^ induced the expression and mitochondrial targeting of the TP53-induced glycolysis and apoptosis regulator (TIGAR [53, 86])—a fructose-2,6-bisphosphatase which prevents the intracellular accumulation of reactive oxygen species (ROS) by increasing the levels of free NADPH and the antioxidant effector, reduced glutathione (GSH), in transduced cells [116–118]. The p30^II^ protein suppressed ROS-production by either c-Myc or the viral oncoproteins Tax and HBZ, dependent upon the induction of TIGAR, and inhibited genomic and mitochondrial DNA-damage and cytotoxicity/apoptosis as a result of the aberrant expression of cellular or viral oncoproteins [53, 86]. These studies further demonstrated that p30^II^ cooperates with c-Myc, Tax and HBZ in cellular transformation/foci-formation assays through the induction of TIGAR, and enhanced the colony-forming potential of these oncoproteins in vitro. As Baydoun et al. [81] have reported that p30^II^ inhibits homologous recombination-directed DNA-damage repair and favors the error-prone NHEJ pathway, it is possible p30^II^ could destabilize the genome and promote the accumulation of somatic mutations that may contribute to viral carcinogenesis. Moreover, HT1080 fibrosarcoma clones expressing the infectious HTLV-1 ACH provirus exhibited higher levels of TIGAR expression associated with reduced oxidative DNA-damage, mitophagy, and apoptosis, as compared to clones that contained a mutant ACH.p30^II^ provirus defective for p30^II^ production [24, 53, 86, 119]. Although the viral transactivator Tax has been reported to inhibit p53 functions [120–122], Zane et al. [106] have demonstrated that Tax does not completely inhibit p53, rather, the p53 protein was shown to contribute to Tax-induced tumorigenesis in *Tax*^+^ *p53*^+/+^ transgenic mice. Wright et al. [42] have also reported that HBZ inhibits p53 functions by inhibiting the p300-dependent acetylation of p53 and recruitment of the p53-cofactor HBO1 to the p21/CDKN1A promoter in transfected cells. However, the HBZ protein induces genotoxic stress and is not highly expressed in vivo [58]; and Billman et al. [49] using RNA-FISH have shown that *tax* and *hbz* are alternately expressed in intermittent bursts in HTLV-1-infected patient cells and observed that many cells do not express *hbz*. It is possible that HBZ could interfere with the p300-dependent acetylation of p53 on lysine residue K372 which is also targeted for methylation by the SET7/SET9 methyltransferases and creates a docking site for the TIP60 chromo-domain for the induction of K120-acetylation and p53-dependent pro-apoptotic signaling [114, 115]. It is therefore likely that p30^II^-interactions with TIP60 may counter the cytotoxicity and oxidative stress caused by viral and/or cellular oncogenes—consistent with the demonstration that p30^II^ cooperated with and enhanced the transforming potential of Tax and HBZ in vitro [53]. p30^II^ could further enhance the lymphoproliferative activity of Tax and/or HBZ by preventing the accumulation of damaging mitochondrial ROS and inhibiting cellular apoptosis induced by these oncoproteins [53, 86]. Recently, Malu et al. [123] demonstrated that p30^II^ prevents Tax-induced genomic instability and mitotic catastrophe induced by NF-κB hyperactivation in the HTLV-1 ACH proviral clone, through the activation of p53 and the p53-dependent repression of Stathmin/oncoprotein-18—a p65^RelA^-binding cofactor and tubulin-destabilizing protein. These findings allude to a possible key ancillary role for p30^II^ and the induction of p53-regulated pro-survival signals in HTLV-1 pathogenesis. In 2018, Romeo et al. [86] demonstrated that HTLV-1-transformed T-cell-lines (MJG11, SLB1, ATL-1, and ATL-7) and primary uncultured HTLV-1-infected ATLL clinical samples contain elevated levels of TIGAR that correlated with oncogenic c-Myc expression as compared to activated hu-PBMCs. Using a highly-penetrant NOD/scid xenograft model of HTLV-1-induced T-cell lymphoma, Hutchison et al. [53] demonstrated that TIGAR is expressed at high levels in engrafted HTLV-1-infected SLB1 or Met-1 tumor lymphocytes, associated with c-Myc dysregulation in the primary tumor masses and infiltrated secondary tissues. The elevated levels of TIGAR in HTLV-1+ tumor cells also correlated with increased angiogenesis and infiltration of the tumor stroma and secondary tissues by murine endothelial progenitors (CD31/Flk1-positive cells); and 2 animals developed splenic hemangiomas associated with HTLV-1-induced T-cell lymphomas [53]. By contrast, there is no evidence that the HTLV-2 p28^II^ synologue possesses transcriptional activity and this functional disparity, taken together with its inability to promote cell-survival, could, in part, account for the different pathogenic properties of HTLV-1 and HTLV-2.

## HTLV-1 p13^II^—a ubiquitinated antagonist of Tax-transactivation

The HTLV-1 p13^II^ protein is a mitochondrial targeting factor, comprised of 87 aa residues, and corresponds to the C-terminus of the p30^II^ sequence beginning with a methionine start codon at position 155 (Fig. 4a, b) [25, 124, 125]. p13^II^ is produced from a singly-spliced *pX*-*orfII*-*p13*^*II*^ mRNA which splices a donor nucleotide at position 119 to an acceptor site at position 6875 [87, 90]. Interestingly, the *pX* region of HTLV-2 does not encode a functional synologue of p13^II^ and this represents a significant point of divergence between these PTLV family members. In 1997, however, Mahieux et al. [126] identified a phylogenetically distinct isolate of STLV-1 (STLV-1*marc1*) from an Asian monkey species, *Macaca arctoides*, which lacked the methionine initiation codons for both p12^I^ and p13^II^ and serologically more closely resembled HTLV-2. The mitochondrial targeting signal (MTS) of p13^II^ spans the amino-proximal residues 20–35 (Fig. 4b) which are predicted to form an amphipathic alpha-helix [124]. In 1999, Ciminale et al. [124], using nested deletions and site-directed mutagenesis, demonstrated that mitochondrial targeting of the p13^II^ protein is atypical and does not require the basic residues within its MTS. p13^II^ is required for viral infectivity and the maintenance of a high proviral titer in vivo, which was demonstrated by experimentally inoculating rabbits with a human B-cell line that contained a mutant infectious clone of HTLV-1 defective for p13^II^ production (729.ACH.p13) [25]. Although the in vivo functions of p13^II^ remain to be completely defined, Andresen et al. [21] have shown that the p13^II^ protein is mono-ubiquitinated on a non-lysine residue and localizes in nuclear speckles in the presence of the viral transactivator Tax and, consequently, interferes with recruitment of the p300 coactivator to Tax-containing complexes and represses transcriptional activation from the HTLV-1 5′ LTR. These findings suggest that ubiquitinated-p13^II^ may help to promote viral latency for the establishment of persistent infections in vivo. The unmodified p13^II^ protein targets the inner membrane of mitochondria, induces membrane depolarization and mitochondrial swelling, opens the apoptogenic permeability transition pore, and results in an increased flux of K^+^ and Ca^2+^ ions and the production of ROS [124, 125, 127–129]. Interestingly, Tibaldi et al. [130] have shown that the proline-rich Src-homology 3 (SH3) domain of p13^II^ (Fig. 4b) interacts with and recruits Src-family tyrosine kinases to the intermembrane space of mitochondria, which resulted in increased mitochondrial tyrosine-phosphorylation and abrogated the physiological effects of p13^II^ upon mitochondrial membranes. In 2004, Silic-Benussi et al. [125] demonstrated that p13^II^ inhibited tumorigenesis and the growth of c-Myc/Ha-Ras-transformed rat embryo fibroblasts, as well as p13^II^-expressing HeLaTet-On cell-lines, in engrafted nude mice. p13^II^ also inhibited cellular proliferation in vitro and caused delayed cell-cycle progression and growth-arrest in nocodozole-treated cells. The p13^II^ protein resulted in an increased sensitivity to C2 ceramide-induced apoptosis as detected by poly(ADP-ribose) polymerase (PARP)-cleavage, and also enhanced the levels of nuclear phospho-CREB in response to Ca^2+^-stimulation in histamine-treated cells [125]. By comparison, the related G4 protein of the bovine leukemia virus (BLV) cooperates with the Ha-Ras oncoprotein and induced tumors in engrafted nude mice [131]; and Lefèbvre et al. [132] have demonstrated that both the BLV G4 and HTLV-1 p13^II^ proteins localize to mitochondria and interact with farnesyl pyrophosphate synthetase (FPPS)—a cofactor involved in targeting oncogenic Ras to the plasma membrane, suggesting there may be some functional overlap between these factors. Further, a mutant BLV proviral clone, defective for G4 production, was impaired in its pathogenic potential and failed to induce leukemia or lymphosarcomas in infected sheep [131]. In 2005, Hiraragi et al. [133] demonstrated that p13^II^ inhibited the growth of Jurkat T-cells at high culture densities, and sensitized these cells to apoptosis induced by either Fas Ligand or ceramide-treatment. The ability of p13^II^ to promote cellular apoptosis was countered by treating p13^II^-expressing Jurkat cells with a farnesyl transferase-inhibitor which prevents the posttranslational modification of the Ras protein and interferes with its membrane localization [133]. Silic-Benussi et al. [127] have further demonstrated that increased ROS production by p13^II^ was associated with the activation of resting primary T-cells which was countered by ROS scavengers, whereas p13^II^ sensitized transformed Jurkat T-cells to apoptosis under conditions of glucose-deprivation. It is thus intriguing to speculate that the ORF-II products, p13^II^ and p30^II^, could act coordinately in HTLV-1-infected cells to promote carcinogenesis—with p30^II^ suppressing ROS-dependent apoptosis by p13^II^ through the p53-regulated induction of the antioxidant effector, TIGAR [53, 86]. The absence of a p13^II^ synologue and dissimilar functions of HTLV-1 p30^II^ and HTLV-2 p28^II^, in terms of their ability to activate cellular pro-survival signals, may, at least in part, account for the different pathogenic properties of these related PTLVs.

## Conclusions

It remains an enigmatic mystery why the HTLV-1 is the only member of the PTLV family that is pathogenic in humans. Both the HTLV-1 and HTLV-2 can infect and immortalize primary T-cells cultured in vitro. Despite its similar genomic organization and structural relatedness, the HTLV-2 is not causally linked with any specific disease, although it has been associated with non-malignant lymphoproliferation and mild neurological symptoms in some infected patients [9, 10, 14]. The major viral transactivator proteins, Tax-1 and Tax-2, exhibit > 77% aa sequence homology and activate CREB/ATF and NF-κB-dependent transcriptional signaling in a nearly identical manner (Fig. 2a, b) [134, 135]. However, the HTLV-2 Tax-2 oncoprotein was observed to be less efficient at transforming rat fibroblasts in vitro [135]; and Semmes et al. [134] have demonstrated that Tax-2 does not induce significant genomic DNA-damage resulting in the formation of micronuclei/microsatellites, as compared to Tax-1 in transfected COS cells.

Similar to other transforming viruses that encode latency-maintenance factors, such as Epstein–Barr virus and the Kaposi’s sarcoma-associated herpesvirus, the HTLVs have developed several strategies to suppress the expression of viral antigens, while simultaneously driving mitotic proviral replication through the activation of cellular proliferative pathways. Indeed, the divergent and dissimilar functions of the *pX*-encoded latency-maintenance factors of HTLV-1 and HTLV-2 may provide clues into the differences in pathogenicity of these PTLVs. The antisense bZIP proteins, HBZ and APH-2, both repress Tax-dependent transactivation and gene expression from the viral 5′ LTR and inhibit NF-κB-signaling through interactions with the p65^RelA^ subunit which prevents its binding to κB-responsive enhancer elements [27, 30, 47, 66, 67, 70]. However, whereas HBZ inhibits AP-1-dependent transcription and modulates FoxP3 and TGF-β-mediated inflammatory signaling, the APH-2 protein activates AP-1 and doesn’t affect TGF-β immunomodulatory signaling. Interestingly, although *hbz* is required for HTLV-1 proviral persistence in vivo, Yin et al. [67] demonstrated that rabbits experimentally inoculated with a 729 B-cell/HTLV-2 proviral clone deleted for *aph*-*2* (∆Aph-2) had higher antibody titers and proviral loads than animals infected with wild-type HTLV-2. These results are somewhat surprising and suggest that HBZ and APH-2 have different roles for the maintenance of viral persistence in vivo, and allude to the potential importance of the other *pX* latency factors: p30^II^, p28^II^, and p13^II^. While the HTLV-1 p30^II^ and HTLV-2 p28^II^ proteins are functionally similar in their ability to negatively regulate Tax-dependent transactivation from the proviral LTR [16–20], unlike p30^II^, there is no evidence that p28^II^ contains transcriptional activity. p30^II^ interacts with the cellular acetyltransferases p300/CBP and TIP60 [18, 20, 84–86], interferes with the recruitment of p300/CBP to Tax/CREB/21-bp-repeat TRE complexes on the HTLV-1 promoter [18], and modulates host cellular gene expression through transcriptional and posttranscriptional mechanisms [19, 53, 78, 84, 86, 101]. In vivo evidence has demonstrated that p30^II^ is required for viral persistence and the maintenance of a high proviral titer—presumably, through the suppression of viral antigens which could help HTLV-1-infected cells evade host immune-surveillance pathways [23, 24]. Alternatively, p30^II^ could enhance the survival of infected T-cells by activating cellular pro-survival genes and antioxidant effectors, such as TIGAR, to prevent the accumulation of cytotoxic metabolic byproducts (e.g., ROS) and counter the oxidative-stress caused by the aberrant expression of viral (Tax and HBZ) and/or cellular oncoproteins [53, 86]. The mitochondrial targeting of the unmodified HTLV-1 p13^II^ protein has been shown to promote the activation of primary T-cells through the induction of low levels of ROS; however, higher levels of ROS, as can be present in oncogenically-transformed cells, induce apoptosis [127]. It is thus plausible that p30^II^, p13^II^, and HBZ may act coordinately to promote enhanced lymphoproliferation and mitotic proviral replication, while preventing the buildup of excessive levels of damaging ROS. The absence of a p13^II^ synologue, together with the disparate functions of APH-2 and p28^II^ relative to their HTLV-1 pX counterparts, could provide a molecular explanation for the weakened pathogenic nature of HTLV-2.

## Data Availability

Not applicable.

## Abbreviations

APH-2: antisense protein of HTLV-2
ATLL: adult T-cell leukemia/lymphoma
CREB: cyclic AMP-responsive element binding protein
p300/CBP: p300/CREB-binding protein
HAM/TSP: HTLV-1-associated myelopathy/tropical spastic paraparesis
HBZ: HTLV-1 basic leucine zipper factor
HDAC3: histone deacetylase-3
HTLV-1: human T-cell leukemia virus type-1
HTLV-2: human T-cell lymphotropic virus type-2
LTR: long terminal repeat
NFAR: nuclear factors associated with double-stranded RNA
NF-κB: nuclear factor kappa light-chain enhancer of activated B cells
NHEJ: non-homologous end-joining
ORF-I/II: open reading frame-I/II
PTLV: primate T-cell lymphotropic virus
ROS: reactive oxygen species
STLVs: simian T-cell lymphotropic viruses
TGF-β: transforming growth factor-beta
TIGAR: TP53-induced glycolysis and apoptosis regulator
TREs: Tax-responsive elements
